# Peripheral Lymphocyte Dynamics in the Immune Microenvironment of Multiple Myeloma During Autologous Stem Cell Transplantation

**DOI:** 10.7759/cureus.102862

**Published:** 2026-02-02

**Authors:** Carlos Agustin Villegas-Valverde, Imilla Casado-Hernandez, Francisco Sotomayor-Lugo, Yandy M Castillo-Aleman, Fatma Abdou, Shadi Sharif-Shamat, Antonio A Bencomo-Hernandez, Yendry Ventura-Carmenate

**Affiliations:** 1 Stem Cells Laboratory, Abu Dhabi Stem Cells Center, Abu Dhabi, ARE; 2 Immunology, Abu Dhabi Stem Cells Center, Abu Dhabi, ARE

**Keywords:** bone marrow transplantation (bmt), cellular therapies, flow cytometry, hematopoietic stem cells (hscs), hsc transplantation, immunotherapy, multiple myeloma, tumor immunotherapy

## Abstract

Background and objective

Autologous stem-cell transplantation (ASCT) remains the standard of care for multiple myeloma (MM), yet it is not curative. Over the past decade, there has been growing interest in immunotherapy-based strategies, highlighting the need for predictive biomarkers to tailor post-ASCT treatment. Characterization of immune status via peripheral lymphocyte immunophenotyping is a critical step in this process. This study aimed to characterize peripheral blood lymphocyte immunophenotypes in MM patients undergoing ASCT at baseline, during stem cell mobilization, and on day +21 post-transplant, and to establish descriptive longitudinal benchmarks that may inform immune monitoring in this clinical setting.

Methods

We conducted a prospective, single-center study at the Abu Dhabi Stem Cells Center (Abu Dhabi, United Arab Emirates (UAE), enrolling all MM patients undergoing ASCT between July 2020 and February 2024 (n = 28), with no additional exclusion criteria. Peripheral blood samples were collected at three time points: before ASCT, on day +21 post-ASCT, and from the apheresis product. Canonical lymphocyte markers were analyzed by flow cytometry using a single, polychromatic 10-color antibody panel on a Navios EX cytometer.

Results

Of the 28 MM patients studied, 40.3% were women. The age range was 23 to 64 years. Markedly low baseline B cell counts were observed (6-175 cells/µL). A significant post-transplant decrease in helper T cells, B cells, and the CD4/CD8 ratio was observed at day +21 compared with baseline. The mean total lymphocyte dose infused (0.20 × 10⁹ cells/kg) was below published thresholds; however, the major lymphocyte subsets exceeded recommended cutoff values, suggesting a potentially favorable prognosis. Dose ranges for non-conventional lymphocyte subsets were also determined

Conclusions

T helper and B cells declined during the peri-transplant period in patients with MM who underwent ASCT, suggesting a lack of association with the autograft dose of homologous populations. This study provides preliminary, descriptive benchmarks for minority immune cell populations, supporting their potential relevance as prognostic biomarkers.

## Introduction

Multiple myeloma (MM) is a hematologic malignancy characterized by the clonal expansion of plasma cells in the bone marrow (BM), which produce monoclonal immunoglobulin, commonly called the M-protein or M band. MM often causes end-organ damage, including anemia, renal dysfunction, lytic bone lesions, and hypercalcemia [1]. MM accounts for approximately 1% of all cancers and 10% of all hematologic malignancies [2]. Its global incidence has increased by 126% since 1990, ranking 21st in overall cancer incidence and 17th in cancer-related mortality as of 2022 [3]. Although the age-adjusted annual incidence in the United States has remained stable at around four cases per 100,000 population, more than 32,000 new cases are diagnosed annually, with nearly 13,000 deaths [4].

Demographically, most MM diagnoses occur in individuals older than 55 years (85%), with 60% diagnosed at or after age 65 [2,4]. The disease is slightly more prevalent in men than in women and is approximately twice as common in African Americans compared with Caucasians [4]. In 2019, Abu Haleeqa et al. published the first review of MM cases in Abu Dhabi, United Arab Emirates (UAE), based on data collected from 2016 to 2018. During this period, 1,582 hospital admissions were recorded with an MM diagnosis per the WHO International Statistical Classification of Diseases and Related Health Problems, 10th Revision (ICD-10) criteria, of which 62 cases were confirmed [5].

Advances in therapeutic regimens, including myeloablative chemotherapy followed by autologous stem cell transplantation (ASCT), have extended median survival in MM to more than six years. Nevertheless, MM remains largely incurable [6]. The tumor microenvironment (TME) in MM plays a pivotal role in disease progression and immune surveillance. It features distinct patterns of cellular infiltration that reflect immune status at the time of transplantation and may influence prognosis [7]. In this context, flow cytometric lymphocyte immunophenotyping provides valuable insights into immune reconstitution throughout the transplant process [8].

Moreover, analyzing the lymphocyte composition of the apheresis product enables quantification of the dose of immune effector cells administered to the patient. The autograft’s cellular profile represents a dynamic balance between antitumor and protumor functions and reflects variable levels of immune dysfunction. Importantly, it is theoretically amenable to manipulation before reinfusion [7,8]. Immunophenotyping in MM is therefore essential for identifying biomarkers that are predictive of treatment response and for personalizing post-ASCT therapeutic strategies. This approach also has implications for the design of novel immunotherapies and translational models that may guide future research directions in MM [9]. Based on this rationale, the present study was conducted to characterize peripheral blood lymphocyte immunophenotypes in MM patients undergoing ASCT at baseline, during stem cell mobilization, and on day +21 post-transplant, and to establish descriptive longitudinal benchmarks that may inform immune monitoring in this clinical setting.

## Materials and methods

Study design

A prospective study design was adopted, which analyzed peripheral immunophenotyping in 28 MM patients undergoing ASCT at the Abu Dhabi Bone Marrow Transplant (AD-BMT) Program of Abu Dhabi Stem Cells Center (ADSCC) from July 2020 to February 2024.

Samples

The samples were from patients who met the criteria for receiving an ASCT after being evaluated at the AD-BMT Program. Three blood samples were collected: the first before ASCT (baseline), the second after mobilization (the day before apheresis), and the third at 21 days. A sample was also collected from the apheresis product.

Flow cytometry immunophenotyping

In this research, the conventional lymphocyte populations were evaluated according to the recommendations of the International Clinical Cytometry Society (ICCS) and the Morbidity and Mortality Weekly Report of the National Center for Infectious Diseases (CDC), as described in Table 1 [10,11]. In addition, non-conventional lymphocyte populations: double-negative T cells (CD4-/CD8-), double-positive T cells (CD4+/CD8+), NKT cells (CD3+/CD56+), and CD56bright NK cells.

**Table 1:** Canonical immunophenotypes* ^*^According to the ICCS Assay Development and Validation of TBNK Lymphocyte Subset Enumeration guidelines and the CDC (Morbidity and Mortality Weekly Report) Guidelines for the performance of CD4+ T-cell determinations in persons with human immunodeficiency virus infection*[10,11] ICCS: International Clinical Cytometry Society; CDC: Centers for Disease Control and Prevention

Cells subtype	Immunophenotype
Lymphocytes	CD45^+^/SSC^low^/CD14^-^
T	CD45^+^/SSC^low^/CD14^-^/CD3^+^
Th	CD45^+^/SSC^low^/CD14^-^/CD3^+^/CD4^+^
Tc	CD45^+^/SSC^low^/CD14^-^/CD3^+^/CD8^+^
B	CD45^+^/SSC^low^/CD14^-^/CD19^+^
NK	CD45^+^/SSC^low^/CD14^-^/CD16^+^/ CD56^+^

**Figure 1:**
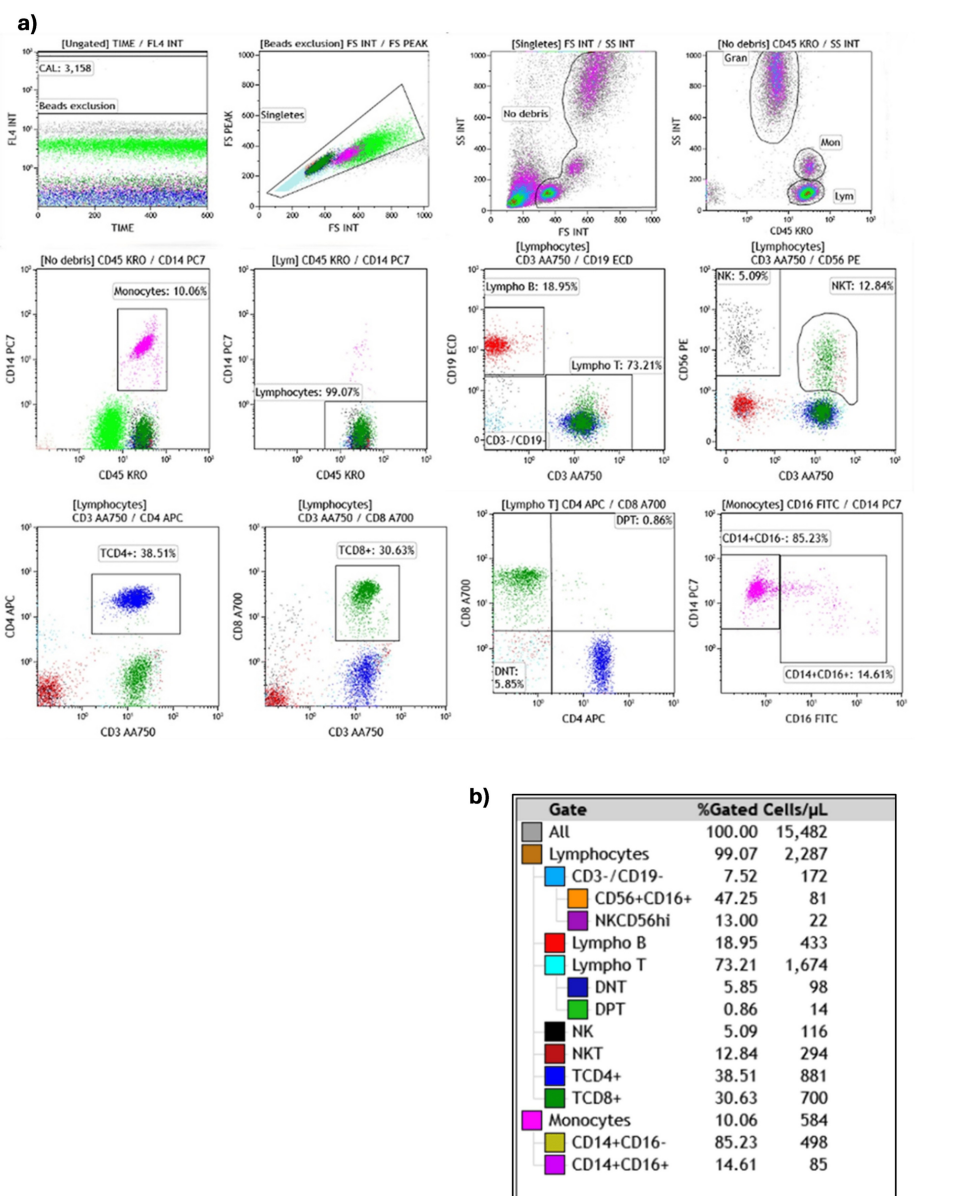
Gating strategies and analysis performed using Kaluza C software with an accurate peripheral blood sample in Beckman Coulter DuraClone IM Basic tubes a) Data analysis with dot plots. b) Gating strategy designed (manual, logic, and sequential) with percentage and absolute count FS: forward scatter (cell size); SS: side scatter (internal complexity); INT: integrated signal (area); PEAK: peak height; TIME: acquisition time; FL4: fluorescence channel 4; KRO: Krome Orange; FITC: fluorescein isothiocyanate; PE: phycoerythrin; PC7: PE–Cyanine 7; ECD: PE–Texas Red; APC: allophycocyanin; AA700: APC–Alexa Fluor 700; AA750: APC–Alexa Fluor 750; CD: cluster of differentiation; Gran: granulocytes; Mon: monocytes; Lym: lymphocytes; CD3−/CD19−: lineage-negative; NK: natural killer cells; CD56+CD16+: conventional NK cells phenotype; NKCD56hi (or NK CD56bright): NK subset with CD56 high expression; Lympho B: B lymphocytes; Lympho T: T lymphocytes (total); TCD4+: CD4⁺ T cells; TCD8+: CD8⁺ T cells; DNT: double-negative T cells (CD4⁻/CD8⁻); DPT: double-positive T cells (CD4⁺/CD8⁺); NKT: natural killer T cells subset; CD14+CD16−: classical monocytes; CD14+CD16+: non-classical monocytes; Cells/µL: cells per microliter

The gating strategy was designed manually, logically, and hierarchically to define the above immunophenotypes and to determine the percentage and absolute counts (Figure 1). Immunophenotyping was performed in a single polychromatic tube, Beckman Coulter DuraClone IM Kit (Cat. #: B53309).

Briefly, 100 microliters of the sample were stained for 15 minutes in the dark at laboratory temperature, then 2 ml of lysis solution was added and incubated for 10 minutes. Just before the acquisition of the sample, 100 microliters of Beckman Coulter FlowCount beads were added. A Navios EX 10-color flow cytometer was used for sample acquisition, and Kaluza C v1.2 software was used for data analysis.

Ethical considerations

The ADSCC Research Ethics Committee (REC) issued approval for this trial in the form of an Exemption Letter (MF-5527-2024-16). All samples and clinical data used in this study are part of routine clinical practice.

Statistical analysis

Descriptive statistics were calculated: absolute and relative frequencies. The Shapiro-Wilk test was used to assess the normality of the distribution of the variables. Due to the asymmetry of the variables, the median was used as a measure of central tendency and the range between the 2.5th and 97.5th percentiles, for the variables referring to blood phenotypes, as it is commonly used in the normality values reported. In the case of phenotypes from apheresis products, the interquartile range was used. The significance threshold was set at p < 0.05.

## Results

Characteristics of the study population

The study cohort included 28 patients with MM, of whom 40.3% were female. The age range for both sexes was 23 to 64 years. Table 2 summarizes demographic characteristics (age, sex) and presents canonical TBNK immunophenotypic values, comparing the study's cohort with healthy adult reference data from other countries [12-17].

**Table 2:** Comparison of main lymphocyte subset values between myeloma patients and healthy adults from different countries ^*^The variables comparison did reveal differences between groups on applying the U-Mann Whitney test with a significance level of ^**^p<0.01 and ^***^p<0.005 ASCT: autologous stem-cell transplantation; Th: T helper cells; Tc: T cytotoxic cells; Bold: mean ± standard deviation; the rest of the lymphocyte subsets are expressed in cells/µL, 95% reference range; ND: not determined; NA: not applicable

Canonical phenotypes	This study (admission)	This study (21 days post-ASCT)	Oman^[12]^	Iran^[13]^	Qatar^[14]^	Turkey^[15]^	China^[16]^	USA^[17]^
Men/women (n)	17/11	17/11	118/NA	150/83	56/54	115/105	77/119	33/67
Age range (years)	23-64	23-64	18-51	20-45	18-55	18-80	20-70	21-67
Cytometer/platform	Navios EX/single	Navios EX/single	FACS Calibur/dual	FACStar/dual	LSR Fortesa/ single	EPICS-XL-MLC/dual	FACS Calibur/single	FACS Calibur/dual
Lymphocytes	650-3023	478-3060	2400-2600	1344-3212	ND	2196 ± 668	ND	2020-2262
T cells (CD45^+^/CD3^+^)	517-2778	439-1765	1612-1790	854-2232	792-3722	725-2960	746-2070	1543-1729
Th cells (CD45^+^/CD3^+^/CD4^+^)	281-1430	191-511**	381-1868	410-1257	546-2591	437-2072	359-1122	942-1065
Tc cells (CD45^+^/CD3^+^/CD8^+^)	101-1151	153-1236	596-679	250-788	375-2532	307-1184	193-976	544-636
B cells (CD45^+^/CD19^+^)	6-175	2-18***	320-377	159-568	43-750	74-586	78-429	231-280
NK cells (CD45^+^/CD3^-^/CD56^+^/CD16^+^)	4-506	4-1194	270-345	81-377	103-1194	4-367	101-708	189-235
Th:Tc rate	0.62-3.97	0.28-1.7*	1.6 ± ND	1.61 ± 0.92	0.4-3.3	1.06-2.76	0.64-3.71	1.7-1.9

At admission, total lymphocyte counts, as well as T cells, cytotoxic T lymphocytes (Tc), and natural killer (NK) cells, ranged from moderately low to within normal limits. These values were comparable to reference ranges reported for healthy adults in the Middle East, Europe, China, and the United States. However, helper T cells (Th) and B cells showed markedly low values, and the Th/Tc ratio was also decreased (Table 2). These alterations persisted through day +21 post-ASCT, remaining close to the lower threshold of normal.

Kinetics of lymphocyte subsets in peripheral blood

**Figure 2:**
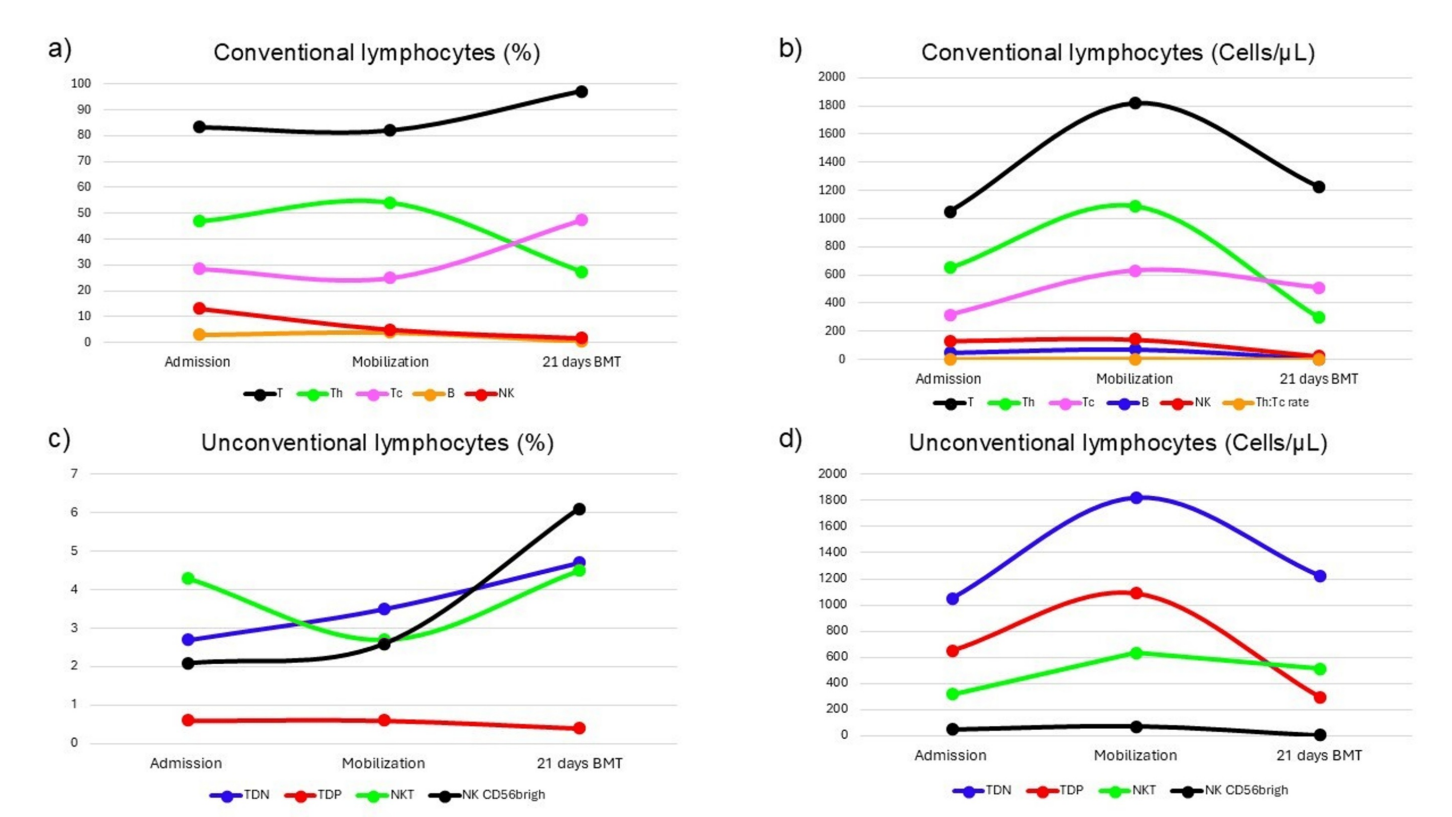
Kinetic profile of peripheral blood lymphocyte concentrations during ASCT a) Relative concentration of conventional lymphocytes; b) absolute count of conventional lymphocytes; c) relative concentration of non-conventional lymphocytes; and d) absolute count of non-conventional lymphocytes. ASCT: autologous stem-cell transplantation; T: T lymphocytes; Th: helper T lymphocytes; Tc: cytotoxic T lymphocytes; B: B lymphocytes; NK: natural killer cells; DNT: double-negative T cells; DPT: double-positive T cells; NKT: natural killer T; NK CD56bright: secretory NK

Throughout the peri-transplant period, conventional lymphocyte subsets displayed distinct kinetic patterns (Figures 2a, 2b). The Th population showed a pronounced decrease in both absolute counts and relative frequency by day +21 post-transplant, without evidence of recovery. B cells remained persistently low in both absolute and percentage terms across all time points.

Tc increased in relative proportion on day +21 (47.3%). Although their absolute count rose during mobilization, it did not substantially modify the total T cell compartment. Total T cell percentages increased steadily from baseline to day +21, ultimately comprising 97.2% of the lymphocyte population. In contrast, absolute counts peaked during mobilization (1821 cells/µL) and declined at day +21 (1223 cells/µL), while remaining within the reference range. NK cells demonstrated a slight downward trend, though this was not statistically significant. The Th/Tc ratio remained low and stable throughout the study.

Unconventional lymphocyte subsets showed dynamic behaviors across the peri-transplant phase (Figures 2c, 2d). T double-negative (DNT) cells increased from 2.7% (1050 cells/µL) at admission to 4.7% (1223 cells/µL) at day +21. NKT cells followed a similar pattern, increasing from 4.3% (316 cells/µL) to 4.5% (511 cells/µL). Double-positive T cells (DPT) maintained relative stability in percentage, though their absolute counts followed a bell-shaped curve, peaking at mobilization and decreasing by day +21 (from 651 to 296 cells/µL). NKT cells, despite representing less than 1% of total lymphocytes, demonstrated a threefold increase in relative frequency by day +21. Their absolute counts, however, remained relatively stable.

CD56^bright NK cells, considered secretory NK cells and typically comprising ~10% of circulating NK cells, showed a notable increase in relative frequency (from 2.1% to 6.1%) during the study period. In contrast, their absolute counts declined sharply (from 47 to 3 cells/µL), indicating a decoupling between frequency and actual cell number.

Immunophenotype of the apheresis product

Immunophenotyping of the autograft revealed that all conventional lymphocyte subsets exceeded established reference thresholds, except for the absolute lymphocyte count (ALC), which averaged 0.2 × 10⁹ cells/kg, below the literature cutoff associated with a favorable prognosis (≥ 0.5 × 10⁹ cells/kg) (Table 3) [18,19].

**Table 3:** Dose of conventional lymphocytes in the infused autograft ND: not determined

Canonical phenotypes	Median dose (cells/kg)	Interquartile range	Cut-off value^18, 19^
Lymphocytes	0.20 x 10^9^	0.14-0.46	≥0.5 × 10^9^ cells/kg
T cells (CD45+/CD3+)	156.7 x 10^6^	94.6-244.0	≥20 × 10^6^ cells/kg
Th cells (CD45+/CD3+/CD4+)	93.7 x 10^6^	52.8-164.4	≥45% of gated apheresis lymphocytes
Tc cells (CD45+/CD3+/CD8+)	51.5 x 10^6 ^	33.4-84.6	>15 × 10^6^ cells/kg
B cells (CD45+/CD19+)	7.7 x 10^6^	3.7-21.2	ND
NK cells (CD45+/CD3-/CD56+/CD16+)	9.4 x 10^6^	2.6-21.3	≥2.5 × 10^6^ cells/kg

By day +21 post-ASCT, 61% of patients had recovered ALC to normal limits, although typically near the lower end of the range. At the time of infusion, only 11% of patients had received autografts with ALC ≥0.5 × 10⁹ cells/kg, whereas 39% achieved >1,000 cells/µL in peripheral blood by day +21 (data not shown).

Non-conventional lymphocyte subsets were also detected in the apheresis product at measurable doses per kg: DNT (5.3 × 10⁶), DPT (0.9 × 10⁶), NKT (5.5 × 10⁶), and CD56bright NK cells (0.2 × 10⁶). These populations were consistently detectable in peripheral blood on day +21 post-transplant, with corresponding absolute counts of 37, 5, 38, and 2 cells/µL, respectively (Table 4). Their presence in both the autograft and recipient peripheral blood suggests their potential involvement in post-transplant immune reconstitution.

**Table 4:** Dose of non-conventional lymphocytes in the infused autograft

Unconventional phenotypes	Autograft median dose (cells/kg)	Autograft interquartile range	Peripheral blood median on +21 day (cells/µL)
DNT cells (CD45+/CD3+/CD4-/CD8-)	5.3 x 10^6^	2.5-7.8	37
DPT cells (CD45+/CD3+/CD4+/CD8+)	0.9 x 10^6^	0.4-2.1	5
NKT cell (CD45+/CD3+/CD56+)	5.5 x 10^6^	2.2-8.4	38
NK CD56^bright ^ (CD45+/CD3-/CD56^ bright^ /CD16+)	0.2 x 10^6^	0.0-0.6	2

## Discussion

MM is currently an incurable disease, and ASCT is a recommended therapeutic strategy for eligible patients younger than 70 years. However, relapse rates remain high after transplantation. A notable epidemiological feature is the predominance of the male sex, a pattern consistent with findings in other studies [1,4,19,20]. According to projections from the American Cancer Society, MM will represent approximately 1.8% of all new cancer cases in 2025, with an estimated 35,780 new diagnoses and 12,540 related deaths in 2024 [20]. The rationale for ASCT lies in the capacity to administer high-dose chemotherapy, enabling the eradication of tumor cells. However, long-term success depends on post-transplant immune reconstitution, which plays a critical role in preventing minimal residual disease (MRD), a key contributor to therapeutic failure. In allogeneic transplants, MRD is countered through the graft-versus-host disease (GvHD) effect, whereas in ASCT, this mechanism is not well understood. It has been suggested that infused autograft cells, beyond CD34+ stem cells, may induce GvHD-like effects without the harmful consequences typically associated with allogeneic hematopoietic stem cell transplantation (HSCT) [18,19].

In MM, the TME comprises hematopoietic stem cells, lymphocytes (B, T, and NK cells), and osteoblasts, among others. These components create a permissive niche that supports disease progression, treatment resistance, and immune evasion [21]. Although several studies highlight the importance of the immune milieu, fewer focus on the dynamics of lymphocyte recovery and the phenotypic characterization of specific immune cell subsets [19,22,23]. To date, the ALC is the only well-established independent biomarker reflecting hematologic recovery after ASCT. In MM, the number of CD4+ and CD8+ T cells, as well as NK cells, has been associated with patient survival [21]. Early lymphocyte recovery has shown a positive correlation with outcomes in plasma cell myeloma and non-Hodgkin lymphoma [22].

A comprehensive review by Zhaoyun et al. compiled data supporting this association, noting that an ALC >1.4 × 10⁹/L was linked to improved overall survival (OS) in newly diagnosed MM patients. Similarly, patients with ALC >0.8 × 10⁹/L on day 29 or ≥1000/mm³ on day +23 had better survival outcomes [22]. A prospective study also indicated early recovery of ALC by day 12 and complete restoration by day 30 post-ASCT [24]. The present study evaluated conventional and unconventional lymphocyte subsets in peripheral blood (PB) at three key time points: baseline, mobilization, and day +21 post-ASCT. Both percentage and absolute values were analyzed, given their distinct biological implications. While a subset of patients reached >1000 lymphocytes/μL by day +21, low CD4+ T cell counts, and an inverted Th/Tc ratio raised concern regarding potential negative effects on OS and event-free survival (EFS). These findings align with prior research linking an abnormal CD4+/CD8+ ratio to disease-related immunodeficiency and poor prognosis [22,25].

Flow cytometry studies evaluating lymphocyte subsets in PB and apheresis products have shown variable results. Although some failed to correlate with survival outcomes, others revealed that higher CD4+ T cell counts and CD4/CD8 ratios were associated with prolonged EFS [25]. Post-ASCT, CD8+ effector T cells often expand more than CD4+ subsets, with a concurrent decrease in regulatory T cells (Tregs), thereby reducing the Treg/CD8+ ratio and perpetuating an inverted CD4/CD8 profile for up to a year [24]. Additionally, a redistribution of T cell subsets has been observed two months post-ASCT, with increased effector memory and late effector cells, CD45RA- Tregs, and Th1 cells, but a decrease in Th17 populations. While these changes lacked direct clinical correlation, patients with higher baseline CD4+ and CD8+ memory T cells showed better five-year outcomes [26]. Notably, elevated gamma-delta (γδ) T cells on day 100 post-ASCT were associated with improved EFS and OS. Likewise, central memory CD4+ T cells post-ASCT were associated with superior two-year OS in MRD-negative patients and those not receiving maintenance therapy [27].

Increased CD19+ B cell counts (>125/μL) have also been associated with better survival in MM [22]. In this study, B cells were markedly reduced at admission, showed slight increases post-mobilization, and remained low post-transplant. These findings mirror those by Chen et al., who reported a baseline median of 89.78 cells/μL and a pre-transplant count of 10.59 cells/μL [28]. Heck et al. documented 95% B cell depletion after induction therapy and conditioning in MM patients [29]. Therefore, the low B cell counts observed at admission may reflect, at least in part, treatment-related effects before transplant, as well as disease-related immune dysregulation. Comparative studies in multiple sclerosis indicate that immune repopulation, particularly of naïve B cells, occurs slowly over one to two years. Delayed B cell recovery has been attributed to both T cell-dependent and T cell-independent disruptions in immune regulation [26]. In terms of innate immunity, NK cells and other mediators typically recover within weeks after ASCT, whereas adaptive immunity lags. NK cell counts below 100 cells/μL one month post-transplant have been linked to significantly lower EFS [22]. Despite early reconstitution of NK and CD8+ T cells, only NK cells have consistently demonstrated a prognostic impact [21].

In this cohort, NK cell counts were within the expected range at admission (100-200 cells/μL) but did not reach commonly reported recovery thresholds by day +21. Notably, the NK content in the autograft was 3.7 times higher than the reference cutoff. Given their critical role in immunosurveillance, these findings suggest that early post-transplant NK recovery may warrant further evaluation, ideally in larger cohorts with extended follow-up and survival-related endpoints. This interest in NK cells has spurred the development of innovative immunotherapies, including CAR-NK cells, immune checkpoint inhibitors, and KIR-targeted approaches [26]. Post-transplant NK cell levels have been linked to early lymphocyte reconstitution and antitumor activity [25,30]. Rueff et al. found that patients with 100-200 NK cells/μL at day +30 had significantly longer EFS (HR = 0.33), and this effect was even greater for those exceeding 200 cells/μL (HR = 0.27) [21].

Natural killer T (NKT) cells have also been recognized for their role in tumor control. Their functional suppression during MM progression is mediated by tumor-derived glycolipids like Gg3Cer, which block antigen presentation [29-32]. Our data showed a twofold increase in NKT cells at day +21, suggesting early immunologic recovery. In MM and other malignancies, unconventional T cell populations such as DPT and DNT cells have demonstrated both regulatory and cytotoxic activity [33,34]. DNT cells have gained attention for their dual ability to kill tumor cells and suppress GvHD. They can be expanded ex vivo from healthy donors, supporting their development as off-the-shelf cell therapies [34-36]. Studies have shown that DNTs release IFN-γ and TNF-α, indirectly suppress host T cell proliferation, and promote leukemic cell apoptosis through Fas/FasL and NKG2D-DNAM pathways. In acute myeloid leukemia (AML) models, DNTs eliminated leukemic blasts effectively, even after chemotherapy [36].

This study has limitations, including a small single-center cohort and limited follow-up (up to day +21 post-ASCT). Therefore, the findings should be interpreted as exploratory and primarily reflective of early immune dynamics rather than long-term adaptive immune reconstitution. Baseline B cell lymphopenia may also be influenced by prior anti-myeloma treatment exposure and other pre-transplant clinical factors. Future studies should incorporate treatment stratification to better contextualize immune reconstitution trajectories in MM. Although detailed immunophenotyping was performed, future studies should include functional immune assays, expanded biomarker panels, larger patient samples, and a longer follow-up to better understand the clinical relevance of immune reconstitution in MM.

Further studies are warranted to enable long-term clinical follow-up of MM patients undergoing ASCT at the ADSCC. These should incorporate survival-related parameters to robustly assess the prognostic relevance of the conventional and non-conventional lymphocyte biomarkers identified in this investigation.

## Conclusions

T helper and B cells decreased during the peri-transplant period in patients with MM who underwent ASCT, suggesting no association with the autograft dose of homologous populations. The descriptive ranges observed for minority lymphocyte subsets and autograft doses in this cohort provide preliminary benchmarks that may inform future studies aimed at identifying immunological biomarkers for clinical monitoring and advancing personalized treatment strategies in MM.
